# Unpacking the Theory Behind One Health Food Safety Programs: A Vietnam Case Study

**DOI:** 10.3389/fvets.2021.763410

**Published:** 2021-12-01

**Authors:** Steven Lam, Huyen Thi Thu Nguyen, Hai Ngo Hoang Tuan, Luong Thanh Nguyen, Hung Nguyen-Viet, Jenny-Ann Toribio, Huyen Le Thi Thanh, Hung Pham-Van, Delia Grace, Fred Unger

**Affiliations:** ^1^Department of Population Medicine, University of Guelph, Guelph, ON, Canada; ^2^Vietnam National University of Agriculture, Hanoi, Vietnam; ^3^Center for Public Health and Ecosystem Research, Hanoi University of Public Health, Hanoi, Vietnam; ^4^Department of Women's and Children's Health, Uppsala University, Uppsala, Sweden; ^5^International Livestock Research Institute, Nairobi, Kenya; ^6^School of Veterinary Science, The University of Sydney, Darlington, NSW, Australia; ^7^National Institute for Animal Sciences, Hanoi, Vietnam; ^8^Natural Resources Institute, University of Greenwich, London, United Kingdom

**Keywords:** One Health, food safety, Vietnam, theory of change, outcome mapping, program evaluation

## Abstract

Many One Health programs are inherently complex, characterized by multiple perspectives from multiple sectors, delivery across various scales, and a focus on complex problems at the convergence of people, animals, and the environment. This complexity makes them difficult to conceptualize, requiring frameworks to organize the different program components. Evaluation frameworks that unpack the sequence of events linking program activities to outcomes (e.g., Theory of Change) and track outcomes (e.g., Outcome Mapping) show promise in supporting the development of One Health programs. While widely used in international development and health contexts, there has been little reflection on the use of Theory of Change and Outcome Mapping within One Health efforts. This paper reflects on the process of applying these frameworks to conceptualize a One Health food safety program in Vietnam. We find Theory of Change fostered the characterization of a change pathway toward safer pork, while Outcome Mapping kept us informed of where along the change pathway we were. One Health programs considering evaluation frameworks should adopt elements that make sense to them, be intentional about co-designing the evaluation, and view evaluation as a process, not a product.

## Introduction

The interaction between humans, live animals for sale, and food products in informal and open-air food markets creates risks for food safety and emerging infectious diseases (1). COVID-19—potentially emerging from markets that sold animals—reinforces the need to prepare for the potential spillover of infections from animal and animal products to humans (2). In drawing attention to multi-disciplinary, multi-sectoral action, the One Health approach is considered a promising strategy to address food safety, animal, and environmental threats (3–5). However, the focus of One Health programs on complex problems at the convergence of people, animals, and the environment, along with the multiple perspectives from different disciplines and sectors, characterize many One Health programs as complex (6, 7).

This complexity makes One Health programs difficult to conceptualize, requiring frameworks to organize the various components of One Health programs (8). Understanding a program's underlying theory is a promising strategy for supporting the planning, implementation, and evaluation of programs, particularly those with multiple interacting components (9, 10). In response to the need to support learning within complex development programming, Theory of Change (ToC) and Outcome Mapping (OM) are receiving growing attention (11, 12). ToC is a tool often used in evaluation for exploring change, how it happens, and why, viewing change processes as dynamic, interlinked, and non-linear (13). OM is an approach to planning, monitoring, *and* evaluation that focuses on social change, placing development actors at the core of its processes (14). Both represent a paradigm shift away from conventional evaluation by focusing attention on what must change *before* considering how change can be achieved.

ToC originated in the context of social change whereby it was difficult to evaluate social change programs that were not clear about what they set out to do and how (15). As its name suggests, ToC is a theory of how and why a program works. While the understanding of ToC has evolved in recent years, ToC is commonly viewed as a critical reflection on a program's strategy, context, and outcomes (16). Increasingly, ToCs are used to facilitate sense-making at regular intervals and are often updated in adaptive programs as new information is learned (17, 18). In contrast, OM is a well-defined approach to evaluation that was adapted from “outcome engineering” (19). OM is designed to support evaluation practitioners in assessing the contributions made by development programs to the achievement of outcomes rather than impact. OM focuses on factors and actors within the program's direct sphere of influence (14).

The shared emphasis on outcomes suggests a common ground for ToC and OM to work together. For example, ToC might provide a shared roadmap toward systems change and highlight potential areas for monitoring and evaluation. However, ToC does not tell us what indicators to monitor, who will monitor them, and when to collect data. OM could facilitate testing and validation of the ToC by analyzing the behavioral changes and interrelationships of development actors. Yet, the operationalization of OM is often resource-intensive, requiring substantial adaptations based on organizational capacity (20). Combining ToC and OM might overcome critiques of each tool and thus be considered a productive endeavor to improve the evaluation of complex interventions.

Although combining the two shows promise in addressing complexity, there are some differences in the theoretical underpinnings between ToC and OM. ToC was developed in response to difficulties in evaluating complex social change programs, calling for the articulation and testing of assumptions underlying change processes (13). Also originating in the context of social change, OM assumes that development happens through behavioral change and that sustainable change requires meaningful engagement with key actors. Given their slightly different histories and research traditions, ToC and OM have developed different practices; ToC focuses on developing a rich description and visual representation of the program theory whereas OM is primarily concerned with understanding or ‘mapping’ behavioral outcomes. While both tools are emerging in development evaluation, there is a paucity of reflective practice on the use of ToC and OM together, particularly in dynamic, low-resource settings (21).

Considering the need for frameworks guiding the conceptualization of One Health programs, and the promising role of ToC and OM, this paper reflects on the experiences of constructing ToC and OM to inform a One Health program. Specifically, the objectives of this paper are to (1) describe how ToC and OM frameworks can be applied to support the monitoring and evaluation of a One Health food safety program in Vietnam; and, (2) reflect on the process, challenges, and opportunities of developing these frameworks. In doing so, we provide lessons in developing One Health food safety programs in dynamic, low-resource settings.

### Context: Addressing Pork Food Safety in Vietnam

We focus on the ‘Market-based approaches to improving the safety of pork in Vietnam’ (SafePORK) program to explore the use of a combined ToC and OM. SafePORK is a 5-year program funded by the Australian Center for International Agricultural Research and implemented by the International Livestock Research Institute, Vietnam National University of Agriculture, Hanoi University of Public Health, and national (National Institute of Animal Sciences) and international partners (University of Sydney). The development of SafePORK was motivated by a growing concern for food safety, one of the most pressing issues among people in Vietnam (22). In particular, the safety of pork is a major concern as pork is the most widely consumed animal source food in Vietnam (23, 24). Pork safety is a shared responsibility among many actors along the pork value chain, making risk management for pork safety a complex challenge. SafePORK operates in several areas of Vietnam (Hanoi, Hoa Binh province, Hung Yen province, and Nghe An province). Applying a One Health approach, SafePORK aims to reduce the burden of foodborne disease in the informal, emerging, and niche markets of Vietnam.

SafePORK can be considered a complex program, characterized by a plurality of stakeholder perspectives and multiple interacting components (25). The research team is comprised of veterinarians, medical doctors, public health experts, farming systems experts, and agricultural economists. They work closely with actors along the pork value chain (e.g., farmers, slaughterhouse workers, wet market retailers, and consumers) and other decision-making partners (e.g., local authorities). Research and development activities of SafePORK often overlap and include generating evidence on feasible approaches; identifying, developing, and piloting light-touch interventions; and, building capacity to manage food safety risks among government partners, private sector actors, journalists, and pork value chain actors. One of the core objectives (number three) of SafePORK is to develop a roadmap showing how, why, and in what context SafePORK leads to safer food (Box 1).

Box 1 **Objectives and activities of the SafePORK program.**

ACIAR Project No. LPS/2016/143

Duration: October 2018 to June 2022

Budget: A$2 Million

**Objective 1:** Generate evidence on the efficacy, feasibility, and reach of current approaches for improving pork safety in Vietnam. Key activities include conducting a rapid value chain assessment, and developing and applying a food safety performance tool.

**Objective 2:** Develop light-touch, incentive-based approaches to food safety. Key activities include selecting five value chains for piloting interventions, establishing a food safety baseline for the selected value chains, conducting participatory research to develop interventions, implementing ‘best bet’ interventions, and evaluating outcomes.

**Objective 3:** Develop a Theory of Change for market-based interventions. Key activities include forming a Food Safety Stakeholder Group, developing a theory of change, and revisiting the theory of change periodically.

**Objective 4:** Support strategies for benefits sharing among men and women in the pig value chain. Key activities include providing gender training, conducting gender analysis of constraints to adopting interventions, and integrating gender considerations into all activities.

**Objective 5:** Build capacity in understanding and managing food safety risks. Key activities include identifying key beneficiaries, providing risk communication training to beneficiaries, disseminating research findings, and evaluating effective communication strategies.

## Methods

### Rationale for Using ToC and OM

Given the complexity of the food safety challenge in Vietnam, the engagement of multiple perspectives characteristic of One Health approaches, and the need for learning support throughout the SafePORK program, we were interested in a framework that was responsive to dynamic, real-world environments. We wanted to systematically capture and learn from our outcomes to inform adaptations to the program. As everyone has different ideas, hypotheses, and assumptions (“theories”) about how change happens, going through a ToC process can help make these theories explicit. We used ToC to establish a shared roadmap toward change and identify potential areas for monitoring and evaluation. A ToC, however, does not tell us how to assess change; we combined ToC with tools offered by OM to support SafePORK in not only learning about its change process but also in measuring it. OM is often considered well-suited to assess programs implemented under complexity in which multiple influences make it difficult to predict what will happen as a program proceeds (26). We used OM to help the team be specific about the actors SafePORK intends to work with, the behavioral changes it hopes to see, and the strategies needed to achieve such changes. Furthermore, we used OM as a framework to monitor outcomes.

### Theory of Change Development and Use

**Figure 1:**
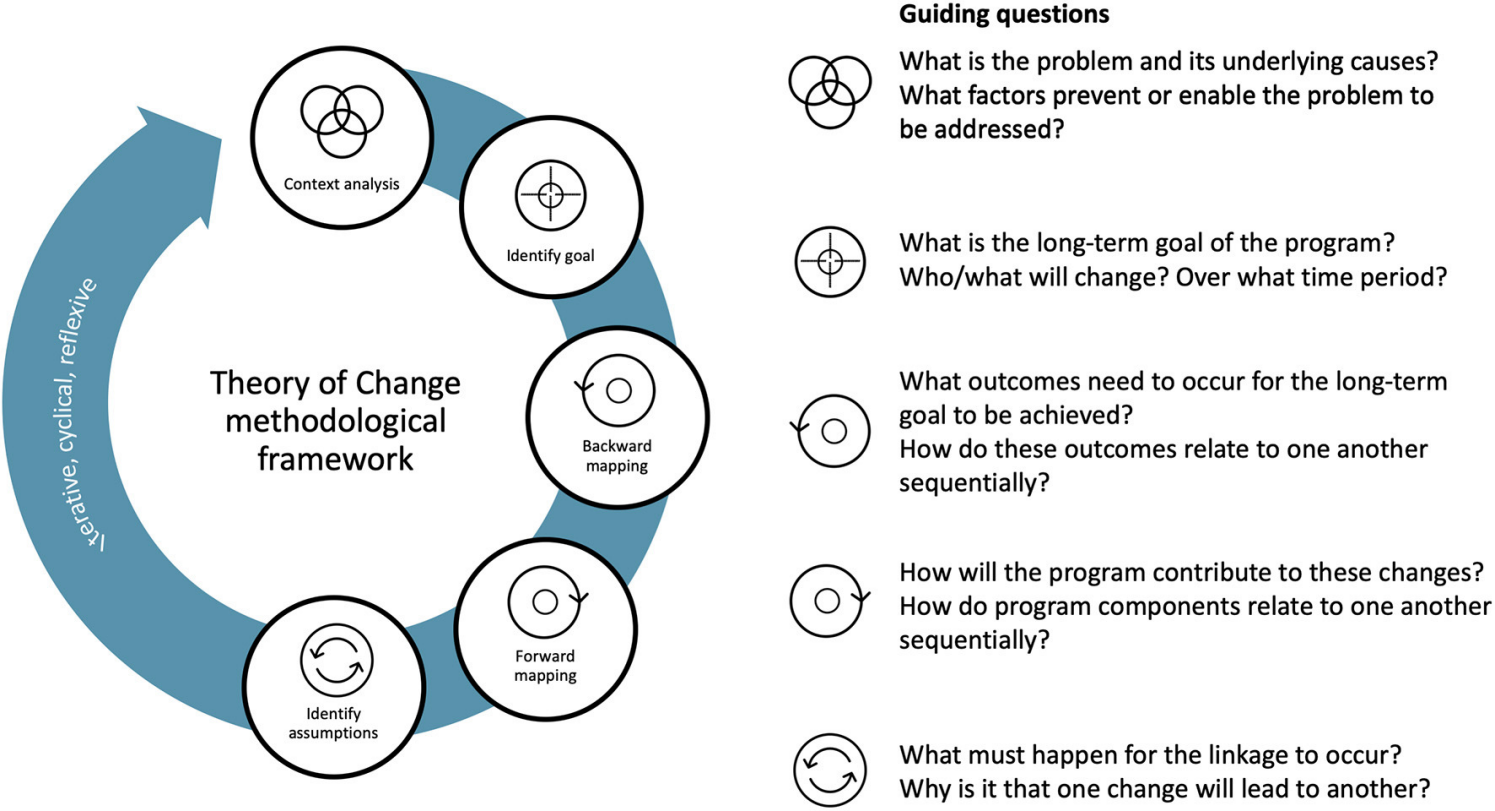
Theory of change methodological framework and guiding questions.

We developed a ToC following advice from several guidance documents (27–29) along with consultations with the team. Often absent from ToC guidance documents is the focus on systems change, yet, capturing systems change is particularly important for food safety programs that influence (and are influenced by) food systems (30). Our adapted 5-step ToC process is iterative, cyclical, and reflective, involving: (1) contextual analysis; (2) identifying the goal; (3) working backward to identify what changes must occur to reach the goal; (4) working forward to identify how the program will contribute to changes; and, (5) stating assumptions underlying change processes (Figure 1). By starting with an overview of potential long-term outcomes at the end of the program (2022), a focus is placed on the bigger picture of systems change. Proposed SafePORK contributions (from 2017 onwards) are added to the change pathway only after systems change is envisioned.

To operationalize the methodological framework for developing a ToC, a full-day workshop was conducted with 12 SafePORK researchers (seven women and five men). The facilitator (SL) described the ToC approach using examples and then asked participants to undertake an exercise following the 5-step process. As SafePORK works extensively with several actors along the pork value chain, participants agreed to create separate actor-based ToCs while acknowledging that ToCs might be combined into one comprehensive ToC later. Participants were split randomly into two teams; one worked on slaughterhouse workers and retailers while another worked on consumers and policymakers. Toward the end of the workshop, participants were asked to reflect on the challenges and opportunities of developing a ToC.

### Outcome Mapping Development and Use

**Figure 2:**
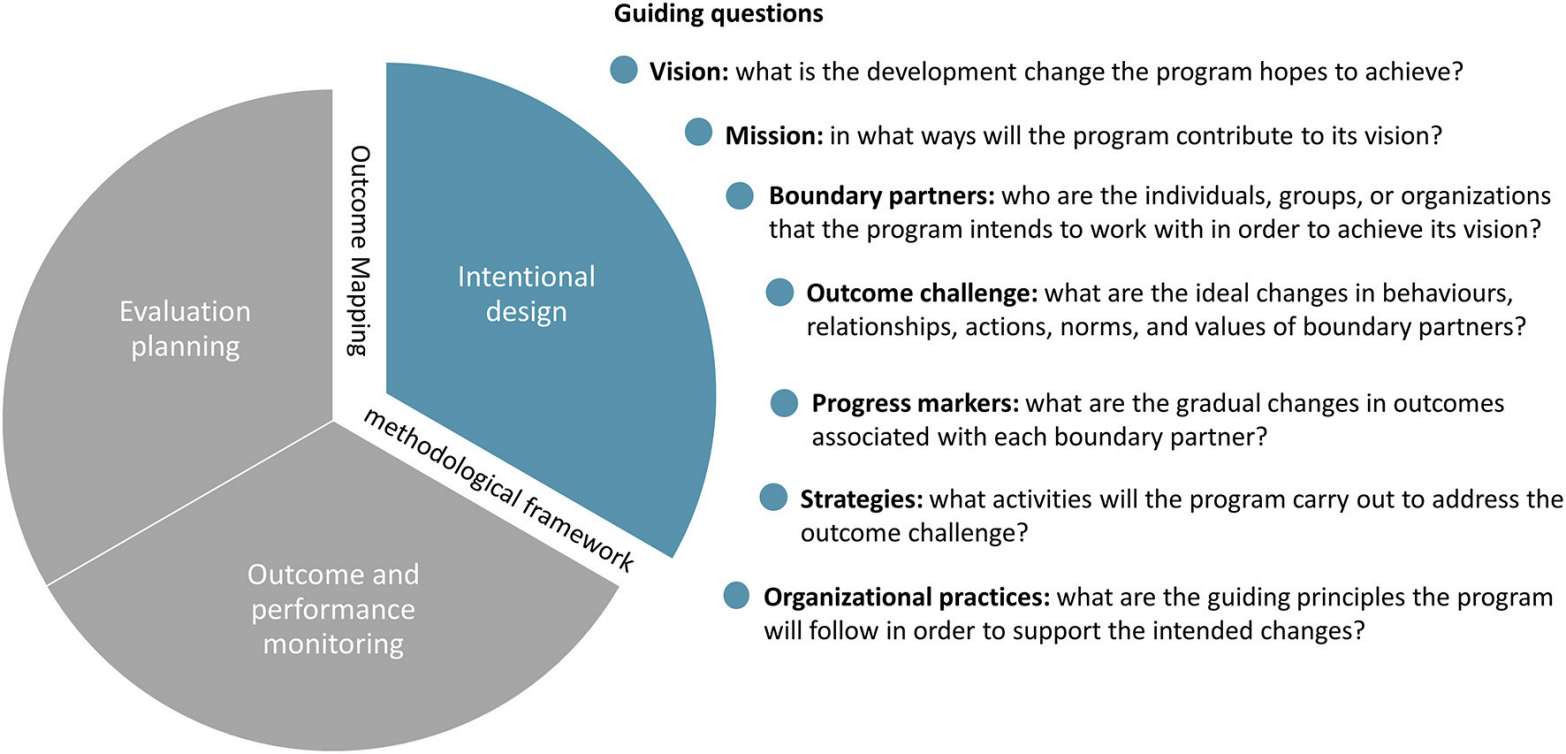
Outcome mapping methodological framework and guiding questions.

OM is a three-stage process of intentional design, outcome and performance monitoring, and evaluation planning (14). In the first stage, stakeholders create a vision of desired behavioral outcomes and outline strategies to be used in achieving such outcomes. The second stage provides a framework for monitoring progress toward changes identified in stage one. The third stage provides a framework for identifying evaluation priorities and conducting an evaluation. To design SafePORK's monitoring and evaluation, we adapted OM; we focused on intentional design to build on the ToC (Figure 2).

To operationalize OM, we convened a half-day workshop with SafePORK researchers (5 women and 4 men). The facilitator (SL) explained the theory of OM and provided examples of OM in practice. As most team members were already familiar with OM through the previous phase of SafePORK (PigRisk program; ACIAR LPS/2010/047; 2012-2017), we worked together quickly through the initial OM steps (i.e., drafting the vision and mission statement). More time was spent focusing on boundary partners, outcome challenges, and progress markers often considered the “essence of OM” (31). Specifically, participants were split randomly into two teams to explore boundary partners, outcome challenges, progress markers, and strategies, and how these relate to SafePORK's vision and mission. Toward the end of the workshop, teams planned for the outcome monitoring.

## Results

### Hypothesizing the Program Theory

**Figure 3:**
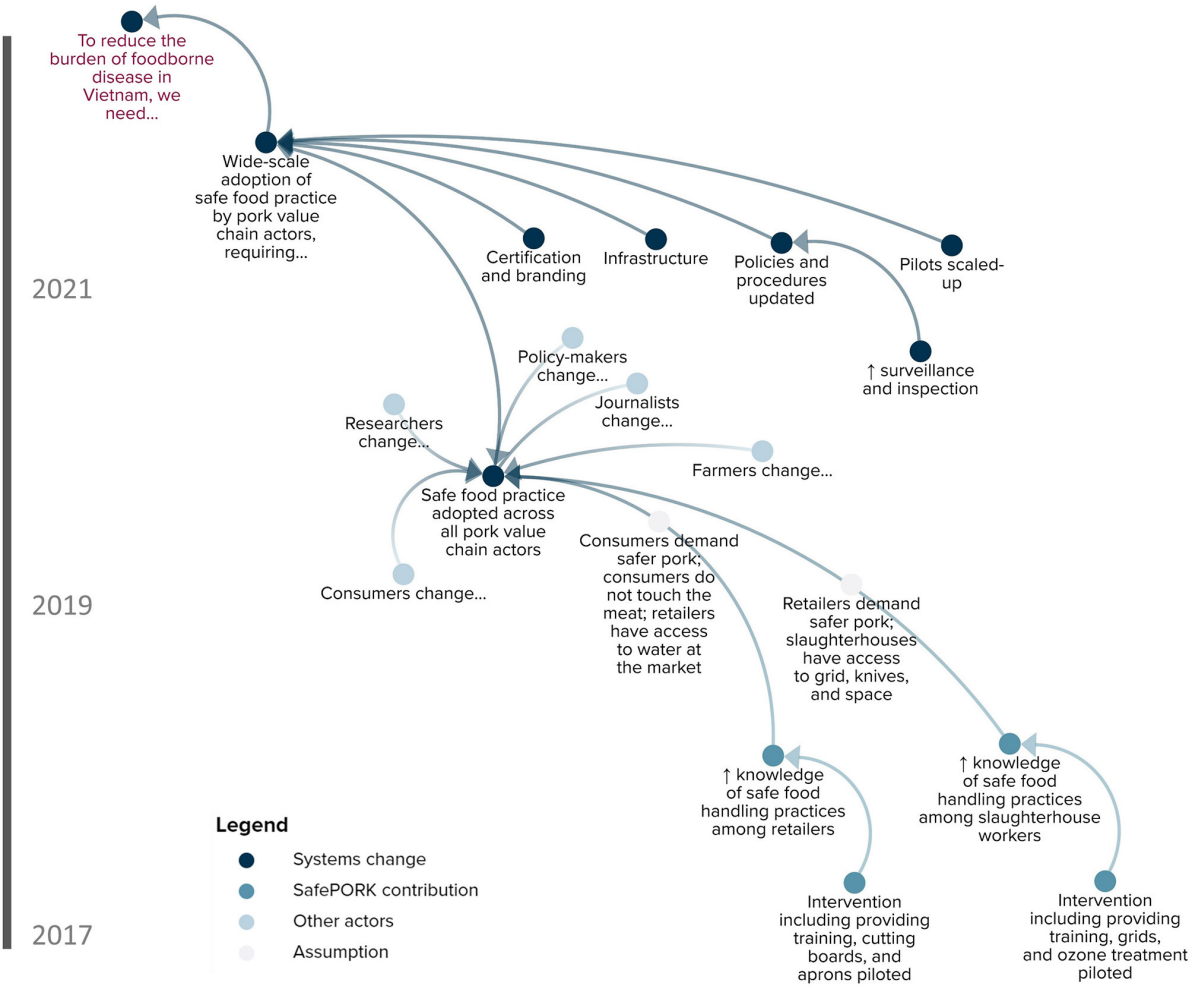
SafePORK theory of change.

The resulting ToC in Figure 3 visually describes the presumed mechanisms of change occurring within the food system in Vietnam. Here, we expand on the ToC by narratively describing the pathway as well as assumptions and context underlying change. The overall goal of SafePORK is to reduce the burden of food-borne disease in traditional, emerging, and niche markets of Vietnam. To achieve this goal, SafePORK proposes that wide-scale adoption of safe food practices among all pork value chain actors is needed. Two pre-conditions are required to achieve this wide-scale adoption: (1) small-scale adoption of safe food practice and (2) updated policies.

According to SafePORK researchers, the identification of cost-effective practices is important for supporting small-scale adoption of safe food practices among women and men. SafePORK intends to contribute to this outcome by piloting light-touch incentive-based interventions, training, and communication along the pork value chain. Several assumptions underlie the causal link between SafePORK activities and improved safe food practices, such as retailers', slaughterhouses', and consumers' demand safer pork. Furthermore, safe food practices need to be supported by broader environmental factors including slaughterhouse and market infrastructure, food safety procedures and enforcement, and certification and branding, which may be indirectly influenced by SafePORK.

Secondly, researchers emphasized that policy-makers should strengthen policies, support the scaling-up of SafePORK pilots, develop a model for small-scale slaughterhouses, improve surveillance and inspection, and increase the budget for food safety interventions. SafePORK intends to influence these actions by presenting evidence from pilot interventions to policymakers through policy brief workshops and study tours. At the provincial level, for example, SafePORK is engaging the sub-Department of Animal Health in Hung Yen in dialogue surrounding the slaughterhouse intervention model. There are several assumptions behind this causal link, such as policy-makers must be interested in improving food safety. While this assumption may seem obvious, the experiences of SafePORK (and PigRISK) demonstrate that buy-in from policy-makers is essential and must be fostered for interventions to succeed.

Researchers also considered the social and physical environments to be important factors underlying the success of SafePORK. For example, Hung Yen is an appropriate province to implement interventions given its high pig production, proximity to the capital city (Hanoi), and room for improvement of hygienic practices. In Hanoi, greater awareness of food-borne diseases, higher income, and generally stronger infrastructure make Hanoi a conducive environment to conduct food safety interventions. Interventions also need to consider who participates in and benefits from efforts aiming to improve food safety and the different roles and responsibilities of women and men. For example, slaughtering is mostly done by men while retailing and purchasing are mostly done by women, providing opportunities for targeted risk management.

### Planning for Monitoring and Evaluation Through Outcome Mapping

**Figure 4:**
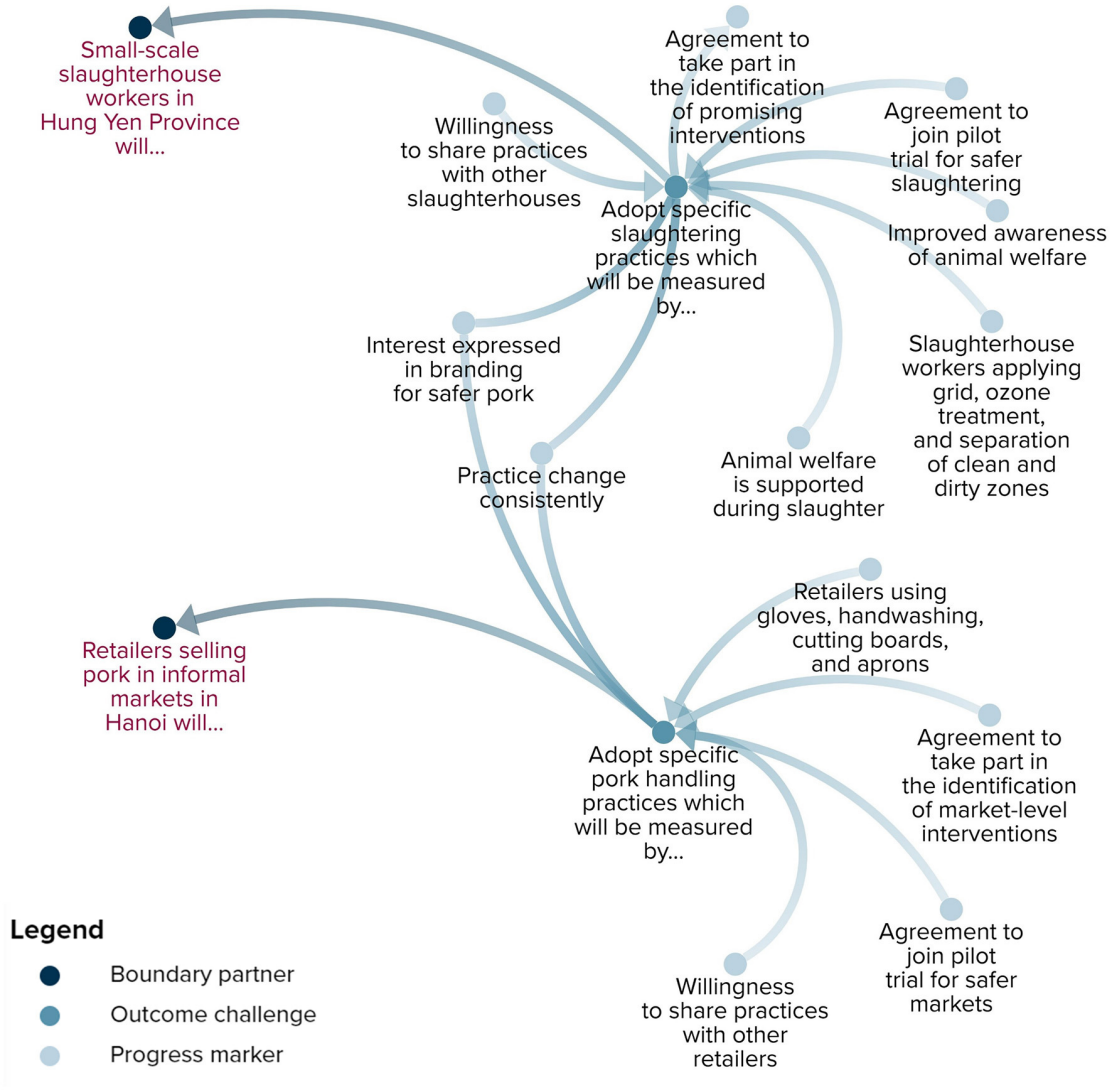
Boundary partners, outcome challenges, and progress markers of SafePORK.

The articulated program theory provided us with a starting point for planning evaluation activities through outcome mapping's intentional design stage. According to SafePORK researchers, the vision of SafePORK is to improve public health by reducing the burden of food-borne disease in traditional, emerging, and niche markets of Vietnam. Its mission is to develop and test market-based, light-touch, and incentive-based interventions. While SafePORK interacts with many boundary partners with a critical role in ensuring food safety, program monitoring and evaluation will focus primarily on slaughterhouse workers and retailers. We consider slaughterhouse workers and retailers to be within SafePORK's direct ‘sphere of influence’ whereas other value chain actors are within SafePORK's indirect ‘sphere of interest’ (14). The main outcome challenge for direct partners is to maintain more hygienic pork handling practices taught in SafePORK training. Progression toward this outcome will be measured by indicators ranging from agreeing to take part in identifying promising interventions to maintaining practice change (Figure 4).

Researchers agreed that the progress of boundary partners toward the achievement of the outcome challenge will be measured on an ongoing, real-time basis. Monitoring journals provided by OM will be used to guide this process. The outcome journal will track the behavioral changes of partners using progress markers whereas the strategy journal will document the activities conducted to achieve outcomes. Several focal points from the SafePORK team will contribute to one shared journal integrating outcomes and strategies. Specifically, the focal points will document (1) activities/strategies implemented, including with whom, where, and when; (2) reflections on what changes occurred, what worked well, and what could be done better; and, (3) and share pictures of before and after. The collected information will be used to inform adaptations to interventions and provide evidence for the final evaluation.

From our ongoing monitoring efforts, we are starting to see behavioral changes during implementation (in some areas and not others), informing adaptations to the intervention. For example, at a slaughterhouse in Hung Yen, we are seeing the provided grid and tables being used during carcass handling. Importantly, some tables were co-invested by the slaughterhouse owner, highlighting the slaughterhouse owner's interest in the program. We are also seeing better separations between clean and dirty areas. However, sometimes knives are not properly cleaned after use and in some cases are put on the floor. The team makes regular visits to the slaughterhouse to encourage hygienic practices. At the traditional wet markets, we are seeing retailers now using separate cutting boards for raw meat and cooked meat. However, many retailers prefer wooden boards because they are better for chopping bones. To address this challenge, the program co-invested in wooden cutting boards with retailers.

## Discussion

This paper describes the experiences of researchers in applying evaluation frameworks to conceptualize a One Health program aiming to improve food safety in Vietnam. We began applying ToC and OM during the formative stages of the program design, enabling us to better anticipate, monitor, and track outcomes early on in the program. We noticed some overlaps and differences between the two outcome-based evaluation frameworks. For example, a ToC focuses on the articulation of a goal, the causal pathways linking short- and medium-term changes to the long-term goal, and the strategies used to achieve outcomes; these steps appear to be consistent with the intentional design stage of OM. However, one notable difference is that ToC emphasizes the need to explicitly define the underlying assumptions behind the change pathways and the contextual factors that influence programming, elements that are typically unexplored in OM (32). Given this complementarity, we agree that combining OM and ToC can be a productive endeavor to support the development of complex programs generally (9, 21, 33) and for One Health programs specifically (9).

### What Is the Promise of Combining ToC and OM?

Combining ToC and OM can address some criticisms associated with each approach. For example, ToCs can sometimes be seen as vague, generic, and simplistic (34). This case study demonstrated that developing progress markers for certain outcome pathways can provide further detail into outcome indicators that are typically missing in ToCs. Conversely, OM is critiqued for neglecting systems thinking by focusing solely on outcomes as behavioral change (35). Yet, OM is generally considered to align with select concepts of systems thinking. For example, *interrelationships* are acknowledged when ‘outcomes’ are defined as patterns of behavior and interactions among stakeholders; *perspectives* of specific actors are accounted for when setting ‘outcome challenges’ for specific actors; and, *boundaries* are considered when selecting ‘boundary partners’, including some actors and excluding others. We consider the interrelationships between stakeholders to be particularly important to monitor; in previous (e.g., PigRISK) and current phases of the program, we considered our partnership as a separate unit of analysis, collecting and sharing reflections about the partnership to ensure various actors operate smoothly as a functioning team (36). Furthermore, because of OM's orientation toward understanding complex and non-linear relationships between different actors that can shape a program, OM is often understood as a complexity-sensitive method (17, 26). To address the critique that OM lacks systems thinking, mapping systems change using a ToC can help to illuminate how boundary partners are influenced by (and are situated within) a social-political system. Furthermore, the progress markers of “expect to see,” “like to see,” and “love to see” reflect the direct response to program inputs and not necessarily a temporal sequence (37); mapping outcomes and their inter-relations in a ToC enables a stronger understanding of when outcomes might occur.

### ToC Then OM, or OM Then ToC: Does Order Matter?

When comparing our process in combining ToC and OM to other programs operating in low-resource settings, we find a variety of processes. We used ToC as a starting point for OM; we kept the findings from the two tools separated to allow for cross-comparisons and to maximize the potential of both tools. In Balls and Nurova, a ToC was created at the program design stage to guide the monitoring and evaluation of sanitation and hygiene research projects in Zambia, Kenya, Malawi, and Tanzania (38). Their ToC illustrated how outcomes will be monitored through OM progress markers, suggesting an effort to combine ToC and OM findings. In other studies, OM was conducted first. For example, OM and ToC were used to encourage 12 non-governmental organizations working on sustainable forest management in Papua New Guinea to align their efforts (39); the ToC was created after the development of OM progress markers to help visualize the relationships between drafted outcome statements. Similarly, in an evaluation of a disaster risk reduction network in the Asia-Pacific region, OM was used to visualize the relationship among stakeholders, the desired behavioral changes, and progress markers; then, ToC was used to identify and test assumptions behind such changes (21). We see the variation in ToC/OM combinations as a strength and a response to the different needs and priorities of programs. We encourage evaluation practitioners to be explicit about their approach in combining the two and to reflect on implications.

### Where Do Conceptual Gaps Remain?

The identification of assumptions underlying the change processes was not found to be particularly difficult, as typically reported in ToC case studies (34); however, one participant emphasized some assumptions were large and require dedicated interventions to address them. A big assumption, for example, is that SafePORK can contribute to the development of food safety certification. Yet, achieving certification is challenging due to short project timelines and the lack of consumer trust surrounding certifications (40). While evaluators have provided clarity on what assumptions are and how to identify them (41, 42), specific guidance is required on whether certain assumptions are better considered as a step along the change pathway or as an assumption underlying the change. In terms of outcome monitoring, we are starting to accumulate a lot of journal entries but struggle in presenting this data in a meaningful way. Some practical examples from the literature visualizing outcome monitoring data would be helpful.

#### Lessons Learned

Three key lessons for evaluation practitioners emerged that are applicable when planning the evaluation of programs operating in dynamic, low-resource settings:

(1) *Adapt tools that make sense to the program and context*. Experimental designs are typically prioritized in evaluations of food safety interventions (43). However, the value of these designs can be limited in environments characterized by complexity. In such cases, this study suggests alternative approaches can be used. We demonstrate how ToC and OM coming from the outcome-based evaluation can be used together toward food safety. For example, the experiences captured in this study show that ToC illuminated potential change pathways while OM, particularly the intention design stage, provided a framework for monitoring progress toward change. These contributions might not have been possible using conventional approaches to evaluation because of the formative nature of SafePORK.
(2) *Be intentional with co-designing the evaluation*. We stress the importance of being intentional about designing the outcome monitoring system. This means providing space and time for team members to come together and think about how the elements of ToC and OM might be combined. It also means working closely with focal points or key members who will collect and share the data. For SafePORK, two focal points made journal entries after each routine visit to the field, which reduced the need for additional human resources and field visits. That said, monitoring outcomes is an additional responsibility for focal points that need to be supported through ongoing training, incentives, and data quality management.
(3) *View evaluation as a process, not a product*. While our team was familiar with OM, developing a ToC was new for some members. We intended to use ToC as a starting point for outcome mapping. The developed change pathway helped to visualize the sequences of and relationships between outcomes. Because it was the first time the team conducted a ToC, we did not expect to have a strong, initial ToC by the end of a one-day workshop. It helped that researchers were made aware of workshop objectives well in advance, creating an environment to participate fully in the exercises. However, when we shared the ToC in a SafePORK planning meeting, it was clear that the ToC could have been further detailed. For example, a researcher who was not able to make it to the workshop suggested that the outcomes are somewhat vague and could be further specified. If we were to do this process again, we would circulate the ToC earlier and on a routine basis. However, we view the ToC as a process, not a product; our next step is to share this initial ToC with our boundary partners for revision, as suggested in Mayne (44). We will continue updating our change pathways as the program proceeds and as new information from outcome monitoring is gained.

### Limitations

We note several limitations to our study. Similar to the experiences reported in Taye et al. (20), we find the progress markers developed may not have been appropriate or realistic. For instance, progress markers for slaughterhouse workers and pork retailers such as “agreement to take part in intervention” might be too simplistic, while “consistent practice change” might be beyond the scope of the project. And like the experiences reported in Balls and Nurova (38), we found some preliminary data from monitoring journals to be messy and inconsistent. Continued reflections by evaluators, researchers, and participants on the development and use of outcome mapping tools would provide important insights to improve evaluation practice. Furthermore, because SafePORK is ongoing, our ToC is a ‘work in progress’; we will keep in mind design considerations, such as a better description of connections (45), to ensure our ToC is testable. Finally, due to resource constraints, our ToC and OM were based on researcher perspectives only. Developing ToC and OM with multiple stakeholder groups along the pork value chain might have led to a more nuanced ToC and OM and a better understanding of priorities to be included. However, through active participation in research and intervention design, stakeholders indirectly contributed to these evaluation activities.

## Conclusion

The challenges and opportunities of frameworks guiding the conceptualization of One Health programs are largely absent from the literature. This study critically reflects on our experiences as researchers in combining ToC and OM during the initial design stages of a One Health food safety program in Vietnam. For the SafePORK program, ToC enabled the scrutinizing of change pathways and the context and assumptions in which change occurs. Equally important, OM provided a framework to help plan and monitor strategies toward and outcomes of safer food. We echo the recommendation in Pasanen et al. when designing outcome monitoring systems: “it doesn't need to be complicated” [(46), p. 30]. Using outcome journals of OM, we are documenting the gradual changes toward steps in the change pathway identified by ToC. While our experiences in using ToC and OM are overall positive so far, we will continue revisiting, revising, and reflecting on our evaluation approach as the program proceeds, contributing to better understandings of pathways toward safer pork in Vietnam.

## Data Availability Statement

The original contributions presented in the study are included in the article/supplementary files, further inquiries can be directed to the corresponding author/s.

## Ethics Statement

Ethical review and approval was not required for the study on human participants in accordance with the local legislation and institutional requirements. The study is a reflection of the authors' experiences in developing a program. No additional information from human participants was collected.

## Author Contributions

SL contributed to conceptualization, research, and writing. All authors read, commented, and agreed on the submitted manuscript.

## Funding

SafePORK was funded by the Australian Center for International Agricultural Research (LS/2016/143) and co-funded by the CGIAR Research Program Agriculture for Nutrition and Health (A4NH).

## Conflict of Interest

The authors declare that the research was conducted in the absence of any commercial or financial relationships that could be construed as a potential conflict of interest.

## Publisher's Note

All claims expressed in this article are solely those of the authors and do not necessarily represent those of their affiliated organizations, or those of the publisher, the editors and the reviewers. Any product that may be evaluated in this article, or claim that may be made by its manufacturer, is not guaranteed or endorsed by the publisher.

## References

[1] Naguib MM Li R Ling J Grace D Nguyen-Viet H Lindahl JF. Live and wet markets: food access versus the risk of disease emergence. Trends Microbiol. (2021) 29 573–81. 10.1016/j.tim.2021.02.007 33712334 PMC9189808

[2] Lin B Dietrich ML Senior RA Wilcove DS. A better classification of wet markets is key to safeguarding human health and biodiversity. Lancet Planet Health. (2021) 5:386–94. 10.1016/S2542-5196(21)00112-1 34119013 PMC8578676

[3] Garcia SN Osburn BI Jay-Russell MT. One health for food safety, food security, and sustainable food production. Front Sustain Food Syst. (2020) 4:1. 10.3389/fsufs.2020.00001

[4] Wielinga PR Schlundt J. Food safety: at the center of a one health approach for combating zoonoses. Curr Top Microbiol Immunol. (2013) 366:3–17. 10.1007/978-3-662-45791-7_238 22763857 PMC7121890

[5] Zhou K Wu B Pan H Paudyal N Jiang J Zhang L. ONE health approach to address zoonotic brucellosis: a spatiotemporal associations study between animals and humans. Front Vet Sci. (2020) 7:521. 10.3389/fvets.2020.00521 32984409 PMC7492289

[6] Destoumieux-Garzón D Mavingui P Boetsch G Boissier J Darriet F Duboz P. The one health concept: 10 years old and a long road ahead. Front Vet Sci. (2018) 5:14. 10.3389/fvets.2018.00014 29484301 PMC5816263

[7] Xie T Liu W Anderson BD Liu X Gray GC. A system dynamics approach to understanding the One Health concept. PLoS ONE. (2017) 12:e0184430. 10.1371/journal.pone.0184430 28877267 PMC5587294

[8] Lebov J Grieger K Womack D Zaccaro D Whitehead N Kowalcyk B. A framework for One Health research. One Health. (2017) 24:44–50. 10.1016/j.onehlt.2017.03.004 28616503 PMC5454183

[9] Rüegg SR Nielsen LR Buttigieg SC Santa M Aragrande M Canali M. A systems approach to evaluate One Health initiatives. Front Vet Sci. (2018) 5:23. 10.3389/fvets.2018.00023 29594154 PMC5854661

[10] Lam S Dodd W Wyngaarden S Skinner K Papadopoulos A Harper SL. How and why are Theory of Change and Realist Evaluation used in food security contexts? A scoping review. Eval Program Plann. (2021) 89:102008. 10.1016/j.evalprogplan.2021.102008 34600337

[11] Thornton PK Schuetz T Förch W Cramer L Abreu D Vermeulen S. Responding to global change: a theory of change approach to making agricultural research for development outcome-based. Agric Syst. (2017) 152:145–53. 10.1016/j.agsy.2017.01.005

[12] Belcher B Palenberg M. Outcomes and impacts of development interventions: toward conceptual clarity. Am J Eval. (2018) 39:478–95. 10.1177/1098214018765698

[13] James C. Theory of Change Review: A Report Commissioned by Comic Relief. London: Comic Relief (2011).

[14] Earl S Carden F Smutylo T. Outcome Mapping: Building, Learning and Reflection Into Development Programs. Ottawa, ON: IDRC (2001).

[15] Weiss CH. Nothing as practical as good theory: exploring theory-based evaluation for comprehensive community initiatives for children and families. New Approaches Eval Community Initiat Concepts Methods Context. (1995):65–92.

[16] Valters C. Theories of Change: Time for a Radical Approach to Learning in Development. London: ODI (2015).

[17] Pasanen T Barnett I. Supporting Adaptive Management: Monitoring and Evaluation Tools and Approaches. London: PRISE (2019).

[18] Floate H Durham J Marks GC. Moving on from logical frameworks to find the “missing middle” in international development programmes. J Dev Eff. (2019) 11:89–103. 10.1080/19439342.2018.1551921

[19] Kibel B. Outcome Engineering Toolbox. Chapel Hill, NC: PIRE (2000).

[20] Taye H Bendapudi R Swaans K Hendrickx S Boogaard B. Outcome mapping as a monitoring and evaluation tool for livestock value chain interventions: the case of imGoats. J Multidiscip Eval. (2018) 14:1–19. Available online at: https://journals.sfu.ca/jmde/index.php/jmde_1/article/view/490

[21] Nirarita E. Combing Logframe With Outcome Mapping (om) & Theory of Change (toc). (2014). Available online at: https://www.outcomemapping.ca/nuggets/combining-logframe-with-outcome-mapping-om-theory-of-change-toc (accessed Apr 28, 2020).

[22] The World Bank. Vietnam Food Safety Risks Management Challenges and Opportunities. Washington, DC: The World Bank (2016).

[23] Dang-Xuan S Nguyen-Viet H Meeyam T Fries R Nguyen-Thanh H Pham-Duc P. Food safety perceptions and practices among smallholder pork value chain actors in Hung Yen Province, Vietnam. J Food Prot. (2016) 79:1490–7. 10.4315/0362-028X.JFP-15-402 28221937

[24] González-Santamarina B García-Soto S Dang-Xuan S Abdel-Glil MY Meemken D Fries R. genomic characterization of multidrug-resistant salmonella serovars derby and rissen from the pig value chain in Vietnam. Front Vet Sci. (2021) 8:705044. 10.3389/fvets.2021.705044 34513973 PMC8429848

[25] Shiell A Hawe P Gold L. Complex interventions or complex systems? Implications for health economic evaluation. BMJ. (2008) 336:1281–3. 10.1136/bmj.39569.510521.AD 18535071 PMC2413333

[26] USAID. SPACES MERL: Systems and Complexity White Paper. Washington DC: USAID (2016).

[27] Taplin DH Clark H Collins E Colby DC. A Series of Papers to Support Development of Theories of Change Based on Practice in the Field. ActKnowledge (2013).

[28] Hivos. Theory of Change Thinking in Practice. The Hague: Hivos (2015).

[29] Jost C Alvarez S Schuetz T. CCAFS Theory Of Change Facilitation Guide. Copenhagen: CCAFS (2014).

[30] Kusters C ten Hove H Bosch D Herens M Wigboldus S. Conference Report: Monitoring and Evaluation for Inclusive and Sustainable Food Systems. Wageningen: Wageningen Centre for Development Innovation (2019). 10.18174/506604

[31] Outcome Mapping Learning Community. Making Outcome Mapping Work: Evolving Experiences from Around the World. Ottawa, ON: OMLC (2007).

[32] Smith R Maureemootoo J Rassmann K. Ten years of Outcome Mapping Adaptations and Support. Ottawa, ON: OMLC (2012).

[33] Ofek Y. Evaluating social exclusion interventions in university-community partnerships. Eval Program Plann. (2017) 60:46–55. 10.1016/j.evalprogplan.2016.09.004 27680984

[34] Lam S. Toward Learning from Change Pathways: Reviewing Theory of Change and Its Discontents. Can J Progr Eval. (2020) 35:188–203. 10.3138/cjpe.69535

[35] Smutylo T. Systems Thinking and Complexity Theory in Outcome Mapping. Outcome Mapping Learning Community. (2014). Available online at: https://www.outcomemapping.ca/nuggets/systems-thinking-and-complexity-theory-in-outcome-mapping (accessed May 27, 2020).

[36] Lam S Barot M Nguyen-Viet H Unger F. Changes in Researcher Capacity in Assessing Food Safety Risks and Value Chains: Insights From PigRisk team. Hanoi: ILRI (2016).

[37] Dyer K. A checklist for Progress Markers. (2014). Available online at: https://www.outcomemapping.ca/nuggets/a-checklist-for-progress-markers (accessed May 27, 2020).

[38] Balls E Nurova N. Outcome mapping and research into use: analysing monitoring data for effective strategies. Dev Pract. (2020) 30:225–67. 10.1080/09614524.2019.1701989

[39] Moxham N. Adapting OM and Theory of Change With Sustainable Forest NGOs in Papua New Guinea. (2013). Available online at: https://www.outcomemapping.ca/resource/om-ideas-8-adapting-om-and-theory-of-change-with-sustainable-forest-ngos (accessed Jan 13, 2021).

[40] Nguyen-Viet H Tuyet-Hanh TT Unger F Dang-Xuan S Grace D. Food safety in Vietnam: where we are at and what we can learn from international experiences. Infect. Dis. Poverty. (2017) 6:39. 10.1186/s40249-017-0249-7 28209208 PMC5314466

[41] Mayne J. Theory of change analysis: building robust theories of change. Can J Program Eval. (2017) 32:155–73. 10.3138/cjpe.31122

[42] Archibald T Sharrock G Buckley J Cook N. Assumptions, conjectures, and other miracles: the application of evaluative thinking to theory of change models in community development. Eval Program Plann. (2016) 59:119–27. 10.1016/j.evalprogplan.2016.05.015 27324286

[43] Young I Waddell L Harding S Greig J Mascarenhas M Sivaramalingam B. A systematic review and meta-analysis of the effectiveness of food safety education interventions for consumers in developed countries. BMC Public Health. (2015) 15:822. 10.1186/s12889-015-2171-x 26307055 PMC4548310

[44] Mayne J. Useful theory of change models. Can J Program Eval. (2015) 30:119–42. 10.3138/cjpe.230

[45] Davies R. Representing theories of change: technical challenges with evaluation consequences. J Dev Eff . (2018) 10:438–61. 10.1080/19439342.2018.1526202

[46] Pasanen T Ambrose K Batool S Soumelong LE Abuya R Mountfort H. Outcome Monitoring and Learning in Large Multi-Stakeholder Research Programmes: Lessons From the PRISE Consortium. London: PRISE (2018).

